# In Favour of Regional Diabetes Day Hospitals

**DOI:** 10.3390/ijerph16132293

**Published:** 2019-06-28

**Authors:** Victoria Barroso, Ascensión Barroso, Ramón Sanguino, M. Isabel Sánchez-Hernández

**Affiliations:** 1 Faculty of Medicine, University of Extremadura, 06006 Badajoz, Spain; 2 Faculty of Economics and Business Sciences, University of Extremadura, 06006 Badajoz, Spain

**Keywords:** diabetes mellitus, day hospital, quality of life, efficiency in public health, cost–benefit analysis

## Abstract

Diabetes mellitus is considered a public health issue worldwide, with a high prevalence. It is a direct cause of death, disability, and high health costs. In addition, it generates a series of complications of variable types and degrees that have frequent negative effects on the quality of life of the people who suffer from it. Efficiency in public health implies a reduction in costs and improvements in citizens’ quality of life. With the twofold aim of rationalizing costs and promoting an improvement in the care of people with diabetes, we propose a project: a Diabetes Day Hospital (DDH) in Extremadura (Spain). This involves a new organizational model which has already been implemented in other European regions, generating satisfactory results. This study includes details on the structure and operation of the DDH, as well as the expected costs. The DDH allows for a proper coordination among the parties involved in the monitoring and treatment of the disease, and reduces the costs derived from unnecessary admissions and chronic complications. Results show that efficiency in the regional health system could be improved and a significant amount of money could be saved.

## 1. Introduction

Diabetes mellitus (DM) is a metabolic disorder of multiple aetiologies characterized by chronic hyperglycaemia with alterations in the metabolism of carbohydrates, fats, and proteins, resulting from defects in the secretion of insulin, in the action of insulin, or both [1]. Diabetes is a complex, chronic illness requiring continuous medical care with multifactorial risk-reduction strategies beyond glycaemic control. Ongoing patient self-management education and support are critical to preventing acute complications and reducing the risk of long-term complications [2]. DM is considered one of the main public health problems worldwide, mainly due to its high prevalence, the high economic cost to the public health system, and the number of premature deaths it causes every year around the world [3].

Currently, numerous investigations are underway to identify the risk factors for diabetes. The risk factors are those aspects of the lifestyle, environment, or genetic traits of an individual that are associated with the onset of a disease [4]. The identification of these factors could allow the development of measures of primary prevention and to delay onset, which could have long-term consequences for public health policies [5]. 

An improvement in the care of DM would help to increase the life expectancy of the people who suffer from it, given that it would reduce the likelihood and frequency of diabetes-related complications and thus reduce the derived costs. For this reason, new healthcare modalities have appeared, such as Day Hospitalization (DH), which has undergone considerable development in recent years, with a significant increase in efficiency of attention to patients previously admitted to conventional hospitals [6].

Until 2009, the Ministry of Health, Social Services, and Equality in Spain registered day hospital posts in a general manner [7]. Given the importance that this type of hospital is acquiring, since 2010 they have been recorded in four subcategories: medical DH (including oncological DH posts), psychiatric DH, geriatric DH, and surgical DH associated with major ambulatory surgery [8]. We believe that in the future new subcategories will be included, such as Diabetes Day Hospitals (DDHs), the use of which is booming in some Autonomous Communities in Spain such as Andalusia and Catalonia.

DDHs ensure better care for patients with DM and an improvement in their quality of life, while avoiding prolonged admissions, reducing emergencies as much as possible, and preventing the complications associated with the pathology. The importance of efficient use of public health resources for regional economic growth and stability and for the well-being of patients has been studied in recent years [9]. 

Nowadays, specific care facilities available for people with diabetes in the region of Extremadura do not exist. They are usually treated in many different places, such as endocrinology clinics, diabetes nurse clinics, primary care physician clinics, primary care nurse clinics, and hospitals. This situation hinders the centralization and coordination of care, causing a greater expenditure of resources, both material and human, and negative consequences for patient health. For this reason, the objective of this paper is to develop a project for the creation of a DDH within the hospital of Extremadura. The main contribution of this work is to determine the advantages of implementing a DDH in a region, both for an improvement in the quality of life and for a reduction in costs.

The rest of the paper is structured as follows. Section 2 describes the current state of diabetes. Section 3 explains the method proposed to perform the project. In Section 4, we develop the project of the DDH, describing its structure, the patient profile, and its operation. Section 5 shows the results of the study. Finally, in Section 6 and Section 7, the discussion is presented and conclusions are drawn.

## 2. Background

This section discusses existing data on the topic under study in the Autonomous Region of Extremadura, in Spain. Later, once the problem statement has been identified, we present the reasons why the DDH is needed in the region.

### 2.1. Epidemiology of Diabetes in Extremadura, Spain

The prevalence of diabetes has increased in recent years. In Spain, the Di@bet.es Study [10] has shown a real prevalence of diabetes in people over 18 years old of 13.8%, with 6% corresponding to undiagnosed diabetes. In 2011, the Ministry of Health and Consumption carried out a National Health Survey where the prevalence of diabetes diagnosed in the 12 months prior to survey was studied in the population over 15 years of age, with a national average of 6.99% [11]. In this survey, a prevalence of 9.38% was found in the population of Extremadura, higher than the national average, where women presented a higher percentage than men [11].

When analysing the trend in recent years of data pertaining to the region of Extremadura, we can observe a growth in the percentage of diabetes declared, rising from 5.9% in 2003 to 9.38% in 2011. Throughout this period, the figures for diabetes declared in Extremadura were higher than those of the national average [12] (ranging from 5.02% in 2003 to 6.99% in 2011).

More recently, according to the estimate made in the Comprehensive Diabetes Plan (PIDIA) of the Autonomous Community of Extremadura 2014–2018 [13], in this region there were around 88,300 people (8.06%) known to have diabetes in 2014.

### 2.2. Complications Associated with Diabetes

As we can see in the records on diabetes of the Ministry of Health and Social Policy of the Government of Extremadura [14], diabetes generates high hospital care activity closely related to chronic complications.

According to the estimate included in the 2014–2018 PIDIA [13], in 2012 there were around 203.58 discharges per 1000 people with diabetes, representing 10.32% of all hospital discharges in Extremadura. In this sense, diabetes is the second most common diagnosis associated with the main diagnosis that produces the highest morbidity, behind arterial hypertension.

In relation to acute complications (hypoglycaemia, ketoacidosis, and hyperosmolar nonketotic coma), there were 3.43 cases per 1000 people with diabetes. Regarding chronic complications, cerebrovascular accident (CVA) and acute myocardial infarction (AMI) were found to be the major cardiovascular complications, with diabetic retinopathy being the microvascular complication, and lower-limb amputations being the main neuropathic and vascular complications [14].

People with diabetes have a risk of cardiovascular disease ranging from two to four times higher than the rest of the population, according to a study published in 2006 by the American Diabetes and Heart Associations [15]. In Extremadura in 2015, 31.22% of hospital discharges after AMI occurred in people with diabetes [14]. In relation to the hospital discharges recorded due to CVA in people with diabetes, no significant variations were recorded, standing at 27.72% in 2015 [14].

Regarding diabetic foot disease (DFD), amputations are sometimes the only treatment due to failure in the prevention or treatment of diabetic foot ulcers. In 2015, a total of 148 non-traumatic amputations were performed in lower limbs of people with diabetes in Extremadura. These amputations represent 61.4% of all those that are performed, with an upward trend [15]. 

Diabetic retinopathy is the most common microvascular complication and the leading cause of blindness in adults [16,17]. In Extremadura, 37.5% of the population with diabetes had diabetic retinopathy in the period 1997–2001 [18]. The evolution of vitrectomies on the Extremadura population with diabetes has followed a downward trend in recent years [14].

Diabetic nephropathy is the main cause of renal failure [19] and occurs in 30–40% of people with diabetes [16]. Data on the prevalence of diabetic nephropathy in Spain are scarce, since only those patients who undergo renal replacement therapy are registered. In the national study PERCEDIME2, a prevalence of 27.9% of any type of chronic kidney disease was found in patients with type 2 diabetes treated in primary care clinics in Spain [20]. In Extremadura, in a study carried out by the Nephrology Service of Infanta Cristina Hospital in Badajoz, an average incidence of chronic kidney disease of 49.7 cases per million population (annual rate) was estimated over the 1990–2006 period in the province of Badajoz [21]. According to the information system for renal patients in renal replacement therapy in Extremadura since 2001 [22], the incidence of these patients has remained similar, ranging from 114 to 152 patients per year. The second most frequent reason for renal replacement therapy in 2016 was diabetic nephropathy, which occurred in 16.46% of cases.

In 2016, the prevalence of renal patients in renal replacement therapy with diabetic nephropathy as primary renal disease was 16.46%. Curiously, this prevalence is higher in the male population, with rates of 69% (158 cases) in males and 31% (70 cases) in females. In addition, the prevalence of diabetic nephropathy for the period 2008–2016 followed an upward trend, especially in men [22].

### 2.3. Diabetes Mortality

The first WHO Global report on diabetes demonstrates that the number of adults living with diabetes has almost quadrupled since 1980 to 422 million adults. In 2016, an estimated 1.6 million deaths were directly caused by diabetes, making it the seventh leading cause of death [23].

The mortality statistics in the region of Extremadura do not really reflect the weight of the disease in the population, since people with diabetes die from one of their chronic complications and on multiple occasions diabetes is not specified as the basic cause of death. Therefore, we need to use a health indicator to measure the impact of the causes of death in a population. In our case, we analysed the potential years of life lost due to diabetes, which studies the premature mortality due to this disease. Each death is assessed by contemplating the years that the affected person has hypothetically stopped living. Since 1990, premature mortality due to diabetes has been declining [24].

### 2.4. Costs Derived from Diabetes

The existing studies on the direct costs of diabetes in Spain have followed different methodologies, which is why very different results have been obtained. These studies have estimated a total direct cost of diabetes between €841 and €5523 million. This created the need to conduct a study to adapt and update this estimate within the framework of the National Health System.

Crespo et al. [25] estimated that the cost of DM for the National Health System amounted to €5809 million (equivalent to 8.2% of all health expenditure of the National Health System) and that the annual cost per patient with diabetes was about €1770. This study [25] also estimated that the hospital cost of DM in Spain was €1934 million (33% of the total cost of diabetes). Regarding diabetes complications, the pathologies that most influenced this cost were cardiovascular disease and peripheral vascular disease, at €521 and €127 million, respectively. The total pharmacological costs, being the most relevant, were €2232 million, representing 38%. Finally, the expenses generated in primary care by patients with diabetes amounted to €1643 million (28%).

Considering all the above we can conclude that health policymakers must seriously consider DDH projects for improving the quality of life of patients as we show below. 

## 3. Method and Procedure

### 3.1. Design

A descriptive-analytical study based on secondary data was carried out. Regarding the development of the project, several documents were used, published in Spain by the Ministry of Health, Social Services and Equality and by the health services of other regions such as the Autonomous Community of Andalusia and the Autonomous Community of Catalonia.

### 3.2. Research Environment and Sample 

The study is focused on the region of Extremadura, more concretely on the public health ecosystem, the Regional Care Program in Extremadura integrated into the Public Health Care System. In Extremadura, in the western part of Spain, the Regional Heath Service is the regional government entity responsible for health care provision in the Autonomous Community. This entity is structured into Central Services and health districts, with dedicated administrative structures, primary care centres and hospitals. We focus on the potential development of a DDH in Extremadura.

### 3.3. Data Collection and Methods

Several documentary sources were used for the location of the bibliographic documents that supported the work developed. A bibliographic search was carried out using the words: diabetes mellitus, epidemiology, classification, costs, mortality, public health, diabetological education, and therapeutic education in diabetes. To obtain the data referring to the morbidity of the disease and its complications in Extremadura, the databases of the Statistics National Institute and the Registry of data on diabetes of the Government of Extremadura were used. Data were taken from the files of the Regional Health Service and related sources in June 2018. 

### 3.4. Data Analysis Method

A cost–benefit analysis (CBA) was used as a systematic approach for estimating the value against the cost of the decision to develop a DDH project in the region of Extremadura as part of the public health policy.

### 3.5. Ethical Considerations

Public health is primarily concerned with the health of the entire population. Consequently, the study was guided by considerations of justice. The paper attempts to foster the development of a DDH project in the region as a question of public health ethics related to providing equal services to all citizens, but the paper also tries to justify the efficiency of the project.

## 4. Development

### 4.1. Diabetes Day Hospital (DDH)

Day hospitalization is assistance in the hospital for a few hours, either for diagnostic purposes, clinical investigations, and/or treatments that cannot be carried out in the outpatient clinic and that do not justify a complete hospital stay [6]. The DDH is a type of functional and high resolution hospital that aims to provide patient care with diabetes in a prompt and agile manner by health personnel in 24 h. It requires adequate coordination with the rest of the units and services in the hospital area and with primary care for adequate patient care [26]. According to the document of Standards and Recommendations of DH of the Ministry of Health and Social Policy in Spain [6], the DDH Unit is specialized since its organization and management must be adapted to the special characteristics of the patients cared for in this type of units and the configuration of the procedures that such characteristics require.

### 4.2. Legal Framework

In Spain, Law 14/1986 of April 25, General Health [27], determines in Article 29.1 that all health centres and establishments, whatever their level, category, or owner, will require administrative authorization for installation and operation, as well as for the modifications that may be established in terms of their structure.

Likewise, Law 16/2003 of May 28, on Cohesion and Quality of the National Health System [28], in Article 27.3, establishes, by Royal Decree, the minimum requirements that must be required in each Autonomous Community for the regulation and authorization of opening and putting into operation health centres, services and establishments.

According to Royal Decree 1277/2003 of October 10, which establishes the general bases on authorization of centres, services, and health establishments [29], for the authorization of a DH it would be necessary to make a modification of the offer of the General Hospital, which must be previously authorized. For this, the guidelines established in Decree 227/2005 of September 27 must be followed, which regulates the procedure and the necessary organs for the application of the quality model and the accreditation of the sanitary quality of the health centres, services and establishments of the Autonomous Community of Extremadura [30].

Following the premises of Decree 227/2005, the Area Manager shall direct the request for the hospital’s modification to the General Director of Planning, Education and Health Quality of the Ministry of Health and Social Policies of the Regional Government of Extremadura, accompanied by the report of results achieved in the process of health self-evaluation. 

Once the request is received, it will be sent to the Accreditation Committee of Health Quality of the Autonomous Community of Extremadura. Then, the General Director will designate an audit team responsible for the external evaluation of the service and the completion of the corresponding report. This report will be sent to the Quality Accreditation Committee and the resolution proposal will be sent to the General Director of Planning, Education and Health Quality. The accreditation resolution will include the degree and type of accreditation obtained.

### 4.3. Structure of the Diabetes Day Hospital: Location and Resources

#### 4.3.1. Location and Structure

The DDH will be located close to the endocrinology outpatient clinics and will depend functionally on the Endocrinology Service. The ideal access would be from the outside, without the need to enter other more complex areas of the hospital. In addition, this space should facilitate the mobility of health personnel and patients and the distribution of materials, in such a way that displacements and consequently loss of time are minimized [6].

According to the document published by the Ministry of Health and Social Policies in Spain [6], DH must follow a design according to the specific characteristics of this type of patients, ensuring at the same time the appropriate conditions of privacy and dignity. According to this line, our proposal for the structure for the unit is described as follows.

- Waiting room—that of the external endocrinology clinics can be used.
- Doctor’s consultation room—the evaluation of the patient with diabetes will be carried out by the endocrine physician.
- Treatment room—this would be the day hospital itself. In this space, the nurse will give the necessary care to the patient with diabetes, carry out the necessary diagnostic tests, and provide individualized diabetological education.
- Group education room—here the nurse will have a specific room where she/he can carry out group sessions for diabetes education.
- Retinography room—it will be necessary to create a space for the retinograph, since the realization of this test requires specific ambient light conditions and concentration on the part of the patient and the nurse.
- Toilets adapted for people with disabilities—one for the staff and one for the patients.
- Warehouse for consumables.

#### 4.3.2. Resources

• Human Resources

The staff of the DDH will have an endocrine physician in the morning shift and two shifts of nurses that will be experts in diabetes education (one nurse in the morning shift and another in the afternoon shift). The attention and follow-up of the patient in the afternoon shift will be carried out by the endocrine physician on duty. 

In a DDH, patient care is more specific when being attended by a doctor specialized in diabetes. It allows better monitoring of the patient and, therefore, greater control of the disease. In addition, access is much faster. The initial diagnosis is oriented to those patients who have been referred by other services or units, usually primary care, for study or consultation. Any doctor could diagnose the disease and refer the patients for specialized treatment and diabetes education, although they do not have any complications.

In Spain, nurses have knowledge of nutrition and dietetics, as these aspects are included in their university studies. Therefore, the nurses will plan diets for the patients in the DDH, and if necessary, they can count on the help of the hospital dietitian, who is available in every hospital and contactable by telephone.

The Public Sanitary System in Extremadura also includes psychologists, who can help to maintain therapeutic adherence. If nurses and/or the endocrine physician detect needs, the patients will be referred to the psychologists.

• Material resources

The material resources required in each of the spaces described above are specified in Table 1 [6,31,32]:

### 4.4. Profile of the Patient

In the DDH, assistance will be offered to patients of any age (paediatrics, adults, seniors) that meet the following criteria [33,34,35,36]:

- Debut of type 1 diabetes mellitus without acute decompensation.
- Scheduling insulin treatment in patients with type 2 diabetes mellitus and of women with gestational diabetes.
- Decompensation in diabetes mellitus requiring specialized treatment but without the need for hospital admission.
- Implementation and monitoring of insulin infusion pumps and continuous glucose monitoring (CGMs).
- Screening and diagnosis of chronic complications of diabetes mellitus.
- Therapeutic education in diabetes (individual and group) that includes:
  o Education about the disease and its complications.
  o How to act in acute complication.
  o Care for control of the disease: carbohydrates and fat intake, foot care, physical and sporting activity, and so on.
  o How to follow the treatment correctly.

### 4.5. Operation of the DDH

#### 4.5.1. Schedule

As indicated in the Comprehensive Diabetes Plan 2007–2012 [33], the DDH will operate uninterrupted for a minimum of 12 h a day, so we propose a schedule from 08:00 h. to 22:00 h from Monday to Friday.

In case the patient must stay on the DDH during meal times, they will be served at the following times: breakfast—09:00 h; lunch—13:30 h, and dinner—20:30 h.

The diets will be requested by the nurse through the corresponding application in the regional software called “Jara”. They will be provided to the unit directly from the kitchen, like the rest of the units, and will be served to the patients by the nursing staff.

#### 4.5.2. Referral to the DDH

Patients who meet the criteria described above will have access to the DDH in less than 24 h from: external consultations, hospital emergency service, primary health care and other hospitalization units/services.

The referral to the DDH from other units/services/level of care will be made through a request for inter-consultation via “Jara” or by telephone. The patient must be accompanied by a report where the doctor indicates the tests and measures carried out.

Once the referral document has been requested and received, it will be the endocrine doctor of the DDH who will call the patient for care. This summons will be communicated by telephone by the nurse of the DDH to the requesting doctor or nursing staff of the hospital service from which the referral is made, or to the patient if coming from primary care.

#### 4.5.3. Coordination between Levels and Information Systems

To achieve an adequate coordination when other services or levels of care share a patient with the DDH, information exchange flows will be established through “Jara”, including the points of view of each of the parties and the actions that are being carried out. Likewise, direct telephone contact between professionals of different levels will be facilitated through a functional telephone consultation scheme in an established schedule (directed mainly to Primary Care), in order to support their therapeutic decisions and increase the degree of resolution to that level [37].

It will be necessary to adapt the already existing process of the DH of the “Jara” information system to the DDH, in such a way that additional information can be entered, such as:

- Diabetological education: including number and content of the sessions received, degree of internalization of knowledge, and skills learned and problems detected.
- Communication between services or levels of care: a space could be created where each of the parties should introduce information on the evolution and the actions that are being performed on the patient, and the opinions or considerations that are deemed appropriate.
- Material delivered: all materials associated with continuous glucose sensors and insulin infusion pumps, glucometer, test strips, and so on. In this way both primary and specialized will control it.

#### 4.5.4. Cleaning and Waste Management

Optimal cleaning and hygiene conditions must be ensured in the facilities, equipment and material of the DDH. The cleaning of the unit will be carried out by the cleaning staff of the external consultation area. As for the equipment material (perfusion pumps, for example), it will be the nurse who, after use, performs adequate cleaning and disinfection of it.

In relation to the management of waste, the DDH must ensure a proper removal and disposal of them, identifying and classifying each items of sanitary waste generated, according to current legislation and protocols established by the hospital.

## 5. Results

The advantages of implementing a DDH in the region of Extremadura are presented as follows in two complementary dimensions: improvement in the quality of life of patients and expected reduction in costs.

### 5.1. Expected Improvement in the Quality of Life of Patients

In the field of regional economics, social injustices expressed in territorial inequalities have long been implicated in cases of poor health. In previous academic works, some evidence [38,39,40,41] suggests that territories defending more socioeconomic justice have higher levels of health than ones that do not. The development of a DDH should be considered a demonstration of such socioeconomic principles in the region. 

Because quality of life is a uniquely personal perception, denoting the way that individual patients feel about their health status and/or nonmedical aspects of their lives, most measurements of quality of life in the medical literature seem to aim at the wrong target. Quality of life can be suitably measured only by determining the opinions of patients and by supplementing (or replacing) the instruments developed by “experts” [42]. According to the previously mentioned percentage of declared diabetes in Extremadura, higher than the national average, after the development of a DDH in the region we could expect some improvements in the quality of life of patients. To this end, we could make a perception survey to measure quality of life of patients. In the near future, the percentage should diminish, at least approaching the national average. 

### 5.2. Expected Savings: Calculation of the Effective Cost of the DDH

For an adequate study of the costs involved in commissioning the DDH, we calculated an estimate of costs. For this purpose, we evaluated the necessary investment to set the DDH up, as well as the related expenses.

Regarding investment, we calculated the cost of remodelling the hospital and the material resources previously shown in Table 2. For the calculation of the quantity of investment imputed annually, we considered a depreciation of 10 years, following the criteria of the Spanish Accounting Rules. Regarding expenses, we calculated: the cost of human resources (including 1.25 doctors and two nurses); the necessary adaptation of the “Jara” software; an average of the costs of the related meals (breakfast, lunch, and dinner); the cleaning service (2 h per day) and the management of waste; the fungible sanitary material, which mainly includes complete analytical material, material for peripheral pathway channelling, glycaemic control, and insulin treatment (including glucometers, test strips, lancets, needles, insulin and all materials for CGMs and insulin infusion pumps); and finally the indirect and unforeseen costs, representing 10% of the previous costs, involving those costs that are difficult to quantify, such as electricity and water, as well as unforeseen costs in general. Table 2 shows a summary of the cost estimate.

### 5.3. Calculation of the Expected Benefits of the DDH

Once the full costs were obtained, we calculated the cost per patient, considering an estimated number of patients of 4417 per year [26] (an average of five new consultations and 18 reviews per day, giving a total of 23 patients per day). The estimated cost per patient of our project was €194.82. This cost should be compared with the cost of stay and bed occupied, €838.98 per day (considering that the stay is to sleep one night and have one of the main meals) [43]. Consequently, we can say that the expected saving after the creation of the DDH should be around €645.16 per patient per day.

Savings to the health care public regional system would be obtained and a better quality of life of related patients would be added to reinforce the economic analysis. 

## 6. Discussion

The main objective of this study has been to develop a project for the creation of a DDH at regional level, as a new organizational form of caring people with diabetes. In this work, we have developed a project focusing on the following aspects:

—Determining how the project will contribute to the efficiency of the Regional Public Health Service, detailing the structure, functioning, and related costs of the DDH. To evaluate the technical efficiency of the DDH project to meet the needs and expectations of healthcare managers and policymakers, a reduction in costs had to be tested.

—Improving the quality of life of patients. The DDH offers an excellent opportunity to improve the care of the person with diabetes inside and outside the hospital environment, offering a better coordination between all the parties involved in the monitoring and treatment of the process of the disease, and thus increasing the quality of life of the patient.

—Reducing the costs derived from unnecessary admissions and chronic complications. It was appropriate to consider whether the benefits of inpatient treatment for diabetes could be achieved in a more cost-effective way. Within the context of general healthcare services, technical efficiency was calculated with reference to the relationship between the resources used and the expected saving per patient as a health output. In line with Lambert et al. [44], we have calculated that the day care for patients implies a reduction in the costs of the resources with respect to inpatient care. 

—Emphasizing the role of nurses as a key element in the care, education, and nutrition of patients. In fact, the DDH will contribute to the specialization of the nursing staff, creating a group of qualified professionals.

## 7. Conclusions

In conclusion, it can be stated that in spite of a potential negative assessment by those responsible for public service investments in the region of Extremadura, the expected saving of €645.16 per patient per day for developing the project is encouraging. The negative assessments may be related to the priorities of public health policies in the region and the opportunity to focus on other existing needs.

Results demonstrate that efficiency in the regional health care delivery system under study could improve and a significant amount of money could be saved. Consequently, the implementation of the DDH will contribute to an improvement in the optimization of resources since unnecessary expenses are avoided and chronic complications are more effectively treated, thereby reducing the costs derived from them. For the near future, it is realistic to expect that the next regional public budget will consider executing the project we have developed in this study.

## Figures and Tables

**Table 1:** Material resources of the Diabetes Day Hospital (DDH).

Space	Equipment
Waiting room	Seating: chairs or benches. Food and beverage machines.
Medical consultation	Modular work table with chest of drawers. Ergonomic chair with wheels. Chairs without wheels. Computer with connection to “Jara” software. Printer. Phone. Closet or shelf for material. Examination table. Digital sphygmomanometer. Phonendoscope. Glucometer. Thermometer. Pulse oximeter. Pupillary lantern. Lower limb Doppler echo. Exploration focus. Water intake for hand washing. Soap dispenser. Paper dispenser. Paper bin. Container of sharp objects. Sanitary consumables: gauze, syringes, needles, lancets. Oxygen and vacuum intakes: includes flowmeter and vacuum gauge.
Treatment room	Modular work table with chest of drawers. Ergonomic chair with wheels. Chairs without wheels. Computer with connection to Jara software. Printer. Phone. Closet or shelf for material. Digital sphygmomanometer. Phonendoscope. Glucometer. Thermometer. Pulse oximeter. Electrocardiogram device. Water intake for hand washing. Soap dispenser. Paper dispenser. Paper bin. Container of sharp objects. Sanitary consumables: gauze, syringes, needles, lancets. Varied material: roof hooks, intravenous pole. Oxygen and vacuum intakes: includes flowmeter and vacuum gauge. Electric bed. Bed tables with adjustable height. Perfusion pumps. Varied medication. Curtains between the beds. Refrigerator. Kits for the exploration of the foot (monofilament 10 g, tuning fork 128 Hz, etc.). Electrocardiograph. Hangers.
Group education room	Large square table. Chairs without wheels. Closet or shelf for material. Materials needed for group education.
Retinography room	Retinograph. Closet or shelf for material. Fungible sanitary material. Medication necessary for the test. Ergonomic chair with wheels. Chair without wheels. Work table. Paper bin.
Toilets	Toilet. Sink. Paper dispenser. Soap dispenser. Toilet brush. Mirror. Hanger. Paper bin. Shower. Closet or locker.
Warehouse	Shelves. Sanitary consumables: gauze, syringes, needles, lancets.

**Table 2:** DDH cost estimate (more detailed information about the costs can be offered by the authors.).

Concept	Description	Quantity
**Investment**	Hospital remodelling	€15,000
	Material resources	€23,655 €
**Expenses**	Human resources	€155,950 €
	Software adaptation	€5000 €
	Patient meals	€66,000 €
	Cleaning and waste management	€6000 €
	Fungible sanitary material	€530,040 €
	Indirect and unforeseen costs	€80,164 €
	Total	€860,520 €
